# Induction of macrophage-like immunosuppressive cells from common marmoset ES cells by stepwise differentiation with DZNep

**DOI:** 10.1038/s41598-020-69690-9

**Published:** 2020-07-28

**Authors:** Hyuma Tsuji, Ryo Otsuka, Haruka Wada, Tomoki Murata, Airi Sasaki, Mizuho Itoh, Muhammad Baghdadi, Erika Sasaki, Ken-ichiro Seino

**Affiliations:** 1 Division of Immunobiology, Institute for Genetic Medicine, Hokkaido University, Kita-15 Nishi-7, Sapporo, 060-0815 Japan; 2 Central Institute for Experimental Animals, 3-25-12 Tonomachi, Kawasaki, Kanagawa 21-0821 Japan

**Keywords:** Regenerative medicine, Allotransplantation

## Abstract

Recent progress in regenerative medicine has enabled the utilization of pluripotent stem cells (PSCs) as the resource of therapeutic cells/tissue. However, immune suppression is still needed when the donor–recipient combination is allogeneic. We have reported previously that mouse PSCs-derived immunosuppressive cells contribute to prolonged survival of grafts derived from the same mouse PSCs in allogeneic recipients. For its clinical application, a preclinical study using non-human primates such as common marmoset must be performed. In this study, we established the induction protocol of immunosuppressive cells from common marmoset ES cells. Although similar immunosuppressive macrophages could not be induced by same protocol as that for mouse PSCs, we employed an inhibitor for histone methyltransferase, DZNep, and succeeded to induce them. The DZNep-treated macrophage-like cells expressed several immunosuppressive molecules and significantly inhibited allogeneic mixed lymphocyte reaction. The immunosuppressive cells from non-human primate ESCs will help to establish an immunoregulating strategy in regenerative medicine using PSCs.

## Introduction

Pluripotent stem cells (PSCs) including induced pluripotent stem cells (iPSCs) and embryonic stem cells (ESCs) provide new opportunities in regenerative medicine to generate grafts for transplantation. Considering that PSCs have the potential to differentiate into all three germ layers, PSCs could theoretically generate all kinds of cell types. Successful generation of various therapeutically relevant cell types, including motor and dopaminergic neurons^1,2^, hepatocytes^3^^,^ pancreatic β cells^4^^,^ cartilages, and cardiomyocytes ^5^ from PSCs have been reported. However, there are still reside immunological problems when using PSCs as a therapeutic cell source. Because ESCs are established from inner cell mass of a blastocyst, and they are allogeneic for almost all recipients, autologous iPSCs have been expected to overcome these obstacles. However, the generation of autologous iPSCs faces some problems,the time and costs needed to assess stability, safety, and efficacy in each case.

Alternatively, the utilization of already-quality confirmed iPSCs established from various donors might be a solution for such problems. Indeed, a pioneer project of establishing a bank of ready-to-use iPSCs with immunological features of "HLA homozygous" background has been launched for future therapeutic use^6^. The effort of HLA-matching for donor–recipient combination would improve the result of allogeneic transplantation. However, even when HLAs are matched, various minor antigens are not compatible in most cases between donor and recipients^7^. Thus, transplantation of allogeneic PSCs-derived tissues still requires immune-suppressive therapy.

Several therapeutic strategies based on treatment with immunosuppressive drugs, including the calcineurin inhibitors, mycophenolate mofetil, steroids, and monoclonal antibodies, such as basiliximab and rituximab, have been developed to avoid the rejection of allogeneic tissues and organs^8^. Although the clinical outcomes of these strategies are satisfactory, various problems have been remained represented by chronic rejection and cumulative side effects, such as increased risks of malignant neoplasia and infection. For these reasons, alternative therapeutic strategies are expected to induce immune suppression that is more specific for transplanted organs.

Cell therapy is one of the promising strategies to control the immune rejection. In previous studies, it has been reported that various kinds of immunosuppressive cells, including immunosuppressive macrophages^9^^,^ regulatory T cells^10^^,^ NKT cells^11^^,^ were effective to promote tolerance in allogeneic transplantation. Indeed, the utility of cell therapy in kidney transplantation and liver transplantation in the clinical situation was reported^12–14^. However, there are few reports about immune regulation by cell therapy in transplantation using PSCs-derived grafts.

Preclinical animal models are important for linking basic research and clinical application. Many researchers have used experimental mouse models for biomedical research. However, there are increasing concerns that some findings obtained from mouse models are being disadvantaged in preclinical studies. The common marmoset (*Callithrix jacchus*) is a New World primate species. It is considered potentially useful as an experimental animal in research fields such as immunology^15,16^, neuroscience^17,18^, and pharmacology^19,20^, because of its size and high genetic similarity with humans^21,22^. Moreover, recently, we have reported the establishment of common marmoset ES cells (CMESCs), iPS cells^23,24^ and transgenic animals^25,26^. Therefore, common marmoset has useful traits for biomedical research and attracted attention as a new preclinical animal model.

Previously, we have reported a concept to develop safe and effective immunosuppressive strategy for regenerative medicine based on the use of PSCs^27–29^. We showed that PSC-derived immunosuppressive cells are effective to block allogeneic T cell proliferation and prolong PSCs-derived graft survival.

In this report, we introduce a culture protocol to differentiate macrophage-like cells with immunosuppressive functions from CMESCs. Although immunosuppressive macrophages could not be induced by same protocol as that for mouse PSCs, we employed an inhibitor for histone methyltransferase, DZNep, and succeeded to induce them. These immunosuppressive cells are effective to block allo-stimulated peripheral blood mononuclear cells (PBMCs) proliferation. The immunosuppressive cells from non-human primate ESCs will help to establish an immunoregulating strategy in regenerative medicine using PSCs.

## Results

### Differentiation of myeloid cells from CMESCs

For differentiation from PSCs to haematopoietic cells, there are at least two methods; with or without feeder cells^30,31^. For mouse PSCs differentiation, feeder cells are commonly used in induction protocols^32,33^, since the widely-used feeder cells are from mouse-origin. However, there is no difference between protocols with or without feeder cells for hematopoietic cell induction efficiency in the case of human PSCs differentiation^34^. As feeder cells appropriate for CMESCs differentiation have not been established, we chose to examine protocols without feeder cells.

**Figure 1:**
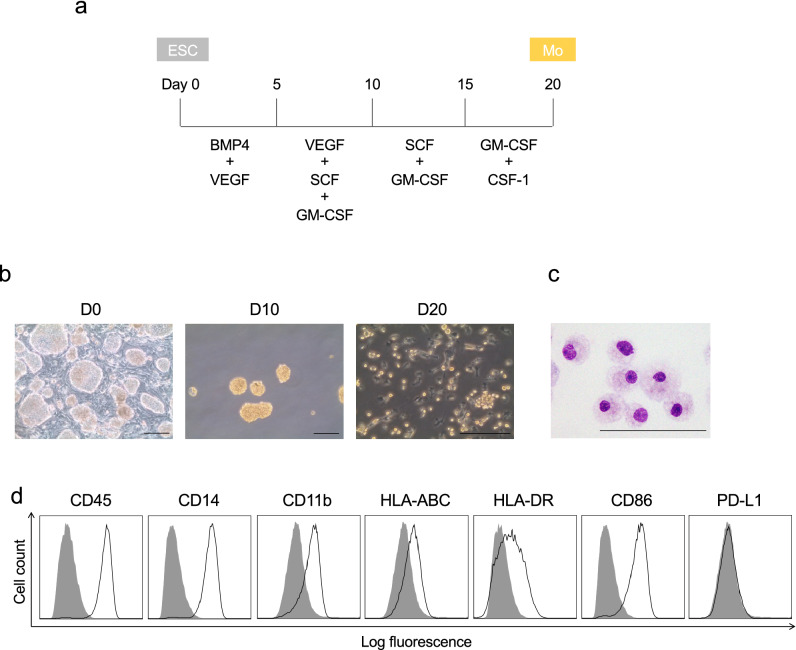
Characterization of CMESCs-derived monocytes. (**a**) A scheme describes the culture protocol used to obtain CMESCs-derived myeloid precursors (monocytes: Mo). (**b**) Morphological change of the cells during differentiation from ES cells to monocytes. Left: transmitted light image of un differentiation ESCs (day 0), Center: EB on day 10, Right: Monocyte-like low adherent cells in day 20. Scale bars: 100 μm. (**c**) May–Grunwald–Giemsa staining of CMESCs-derived monocyte-like cells in day 20. Scale bars: 100 μm. (**d**) Flow cytometric analysis of cell surface molecular expression on monocyte-like cells in day 20. Histogram: gray—sotype control, black line—specific antibody. Data are shown as representative of three independent experiments.

Referring to a previous report of dendritic cell differentiation from human ESCs^31^^,^ we established a protocol for haematopoietic cell differentiation from CMESCs without feeder cells (Fig. 1a). To induce mesoderm and haematopoietic lineage, we used human bone morphogenetic protein (BMP) 4 and human vascular endothelial growth factor (VEGF). At day 5, For cell number expansion, and haematopoietic differentiation, human stem cell factor (SCF) was added. Additively, human granulocyte macrophage-colony stimulating factor (GM-CSF) was used for myeloid lineage direction. At day 10, we could obtain a large number of embryoid bodies (Fig. 1b). At day 20, several cells were lightly attached to petri dish, and others were floating and forming clusters. Morphologically, nucleus was kidney-shaped, and high cytoplasm to nucleus ratio, characteristic of myeloid cells^35^ (Fig. 1c). Then, we examined the cell surface molecule expression of differentiated cells with flow cytometry. These cells were gated on the larger cell population by forward and side scatter, and showed expression of HLA-ABC, HLA-DR, CD45, CD14, and CD86. Additionally, these cells expressed low levels of CD11b. Thus, it was suggested they were monocyte-like cells (Mo). However, Immunosuppressive molecules, PD-L1 was not detectable (Fig. 1d).

### Induction of macrophage-like immunosuppressive cells from CMESCs

**Figure 2:**
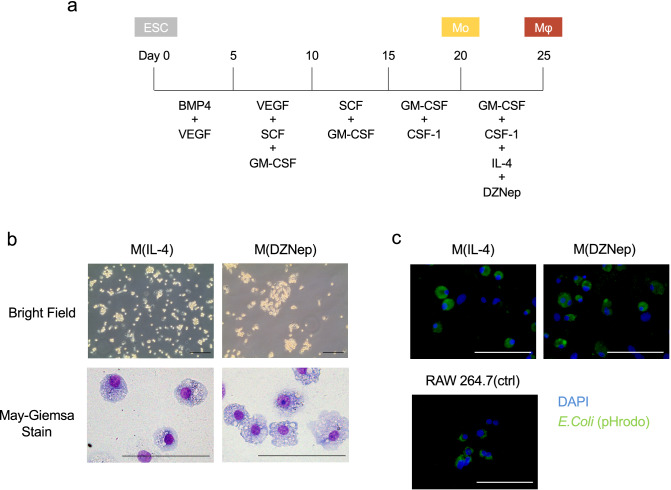
Generation of macrophage-like immunosuppressive cells. (**a**) A scheme describes the culture protocol used to obtain CMESCs-derived macrophage-like immunosuppressive cells. (**b**) Transmitted light image and May–Grunwald–Giemsa staining of CMESCs-derived macrophage-like cells. Scale bars: 100 μm. (**c**) Phagocytosis assay for pH-sensitive pHrodo green *E. coli* bioParticles on indicated cells. Scale bars: 100 μm.

To induce macrophage differentiation from the monocyte-like cells derived from CMESCs, we further cultivated them with human macrophage-colony stimulating factor (CSF-1). As the acquisition of immunosuppressive capacity for macrophages requires Th2 cytokines^36^^,^ IL-4 was added (Fig. 2a). Under the cultivation in the presence of CSF-1 and IL-4, cells differentiated into macrophage-like cells (M (IL-4)) which adhered strongly to the petri dish and contained a phagosome-like structure in their cytoplasm (Fig. 2b, left).

To glance whether the differentiated cells had the immunosuppressive capacity, we examined by mixed lymphocyte reaction (MLR) using mouse T cells. Specifically, we added the M(IL-4) into MLR between CD4 T cells from C3H/He mice and bone marrow-derived DC (BM-DC) from BALB/c mice (Supplementary Fig. 1a). In this experiment, we did not observe the immune suppressive effect of M (IL-4) (Supplementary Fig. 1b). Furthermore, in our previous studies using mouse PSCs, we have reported that addition of lipopolysaccharide (LPS) in the last phase of the culture enabled the cells to acquire NO-dependent immunosuppressive function^27,28^. Therefore, we investigated whether immunosuppressive cells could be induced from CMESCs in the same method (Supplementary Fig. 1a). The addition of LPS at day 20 in differentiation culture induced firmly adherent cells to bacteriological petri dish similar to mouse PSCs (Supplementary Fig. 1b). However, unlike mouse PSCs, NO was not detected in the culture supernatants of LPS-treated CMESCs-derived cells (Supplementary Fig. 1c). Thus, we judged that these cells had no immunosuppressive ability and decided to choose another protocol.

Recently, it has been reported that several low molecular weight compounds which can induce epigenetic changes could enhance differentiation of immunosuppressive macrophages^37,38^. Then, we examined whether such a low molecular weight compounds could induce the immunosuppressive function in macrophages derived from CMESCs. From day 20 of differentiation, we added 3-Deazaneplanocin A (DZNep), a low molecular weight compound which could inhibit histone methyltransferase EZH2, to the culture with CSF-1 and IL-4 (Fig. 2a). After this process, we obtained the cells which showed M(IL-4)-like morphological features (M(DZNep)). M(DZNep) exhibited phagosome-like structure in their cytoplasm and high cytoplasm to nucleus ratio (Fig. 2b, right). Then, we examined the macrophage-like features of M(IL-4) and M(DZNep). Both cells showed comparable phagocytosis activity to mouse macrophage cell line: RAW264.7 (Fig. 2c). To further analyze the M1 or M2 polarization of M(DZNep), we assessed the gene expression of M1 markers such as *irf5*, *il23a*, and M2 markers such as *irf4*, *gata3*^35^. As a result, M(DZNep) showed the expression of M2-polarized marker genes but not M1 markers (Supplementary Fig. 1d).

To assess the suppressive capacity of obtained M(DZNep) in vitro, we next examined whether M(DZNep) could interfere with allogeneic immune response in mouse MLR. We found that the addition of M(DZNep) significantly inhibited mouse T cell response, compared with M(IL-4) (Supplementary Fig. 2a, b, and c). The ability of M(DZNep) to suppress T cell response was further confirmed by serial dilution of M(DZNep) in MLR culture. We found that suppression rate was correlated with the number of M(DZNep) in MLR, which suggests the dose-dependency of the suppressive effect of the cells (Supplementary Fig. 2d).

### Phenotypic analysis of CMESCs-derived immunosuppressive cells

**Figure 3:**
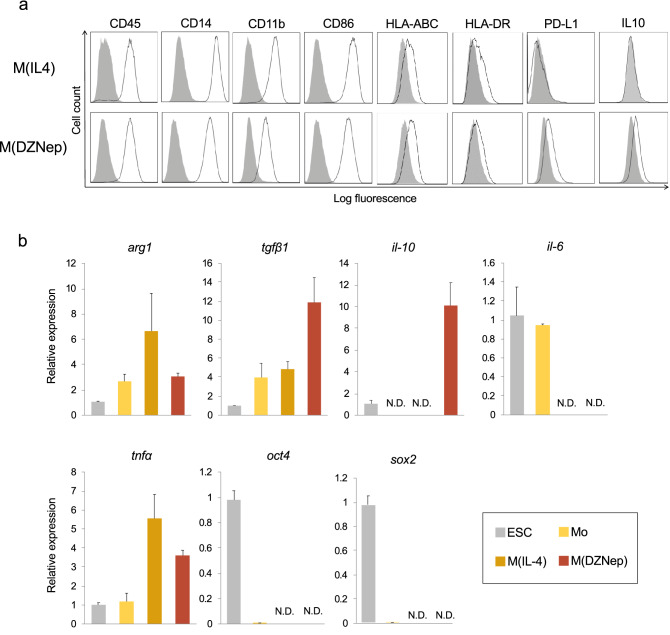
Characterization of CMESCs-derived macrophage-like cells. (**a**) Flow cytometric analysis of cell surface molecular expression on M(IL-4) and M(DZNep). Histogram: gray—isotype control, black line—specific antibody. (**b**) qRT-PCR analysis for expression of macrophage—and macrophage-related genes in M(IL-4) and M(DZNep). Values were normalized to β-actin and shown as mean ± SD from three experiments were shown. ESC = 1, **P < 0.01, *NS* no significance, *N.D.* not detected.

We examined cell surface molecule expression of M(IL-4) and M(DZNep) with flow cytometry. As indicated in Fig. 3a, both cells expressed HLA-ABC, CD45, CD14, CD11b, and CD86 suggesting that they were macrophage-like cells. Intermediate levels of HLA-DR (major histocompatibility class II) were indicative of a partially matured antigen presenting cells. Importantly, M(DZNep) but not M(IL-4) showed expression of an immunosuppressive molecule, PD-L1 (Fig. 3a).

Next, we examined gene expressions with real-time qPCR. An immunosuppressive gene, arginase-1 (*arg-1*) expressed comparably between M(IL-4) and M(DZNep), whereas transforming growth factor-β1 (*tgfβ1*) was higher in M(DZNep) (Fig. 3b). Expression of another immunosuppressive gene, interleukin-10 (*il-10*), could not be detected in Mo and M(IL-4). However, it was observed in ESCs as reported previously^39^ and upregulated approximately 10 times higher in M(DZNep) than in ESCs (Fig. 3b). The expression of IL-10 at protein level was confirmed by intracellular staining with flow cytometry (Fig. 3a). Regarding inflammatory cytokines, interleukin-6 (*il-6*) expression was not detected in both M(IL-4) and M(DZNep), and tumor necrosis factor-α (*tnfα*) expression was not significantly different between both cells. Furthermore, expression of undifferentiated state markers, octamer-binding transcription factor 4 (*oct4*) and sex determining region Y-box 2 (*sox2*) were higher in ESCs, lower in Mo, and not detected in M(IL-4) and M(DZNep). These results suggested that M(DZNep) cells exert their immunosuppressive capacity by the expression of PD-L1, IL-10, and TGF-β.

We next set out to explore the possible mechanisms of M(DZNep)-mediated immune suppression by blocking PD-L1 and IL-10 whose protein expression was confirmed by flow cytometry (Fig. 3a). To inhibit PD-L1 and IL-10 activity, we used anti-human blocking antibodies with validated cross-reactivity to common marmoset corresponding molecules. However, the suppressive effect of M(DZNep) was not canceled by either anti-PD-L1 or IL-10 antibody (Supplementary Fig. 2e). Given that M(DZNep) also showed *tgfβ1* gene expression, the suppressive effect of M(DZNep) may be brought by tgfβ protein. Unfortunately, other inhibitory antibodies to common marmoset immunosuppressive molecules, including anti-tgfβ, are not literarily validated or commercially available. Thus, further pursuit of M(DZNep) effect in immune suppression needs other approaches such as gene knockout or protein knockdown.

### M(DZNep) are capable of suppressing allogeneic immune responses

**Figure 4:**
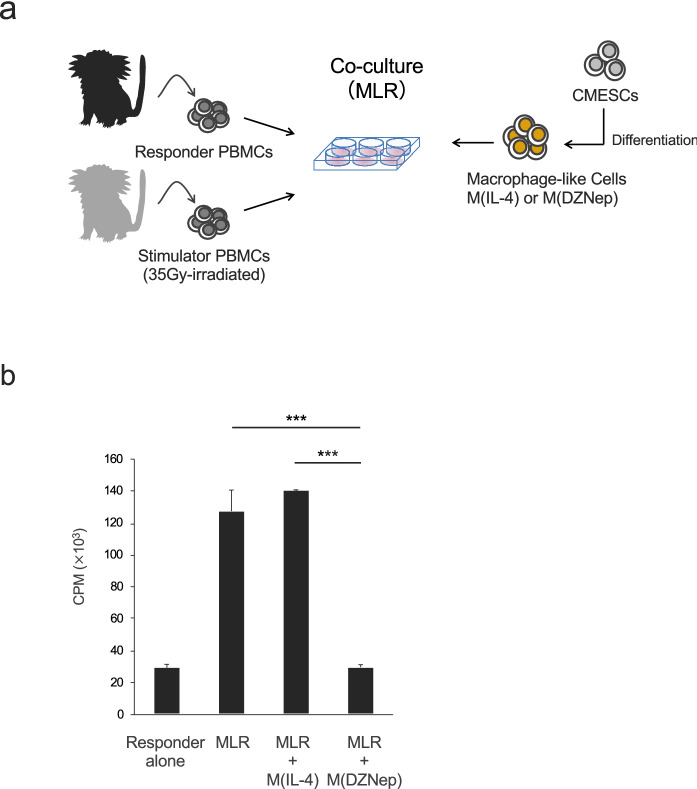
M(DZNep) suppress alloreactive PBMCs proliferation. (**a**) CMESCs-derived M(IL-4) and M(DZNep) were cultured together with two distinct common marmosets PBMCs for 4 days. PBMC proliferation measured for uptake of [^3^H] thymidine. (**b**) MLR assay: Responders (1 × 10^5^) were co-cultured with 35 Gy-irradiated stimulators (Allogeneic common marmoset PBMC, 2 × 10^4^). To test the capability to inhibit PBMC proliferation, irradiated M(IL-4) or M(DZNep) were added to MLR culture at same numbers of stimulator cells. Results are expressed as mean cpm ± SD. ***P < 0.001.

The expression of immunosuppressive molecules, PD-L1, TGF-β, and IL-10 in M(DZNep) raised the possibility that these cells might possess immunosuppressive functions. Thus, we next examined whether M(DZNep) could suppress common marmoset lymphocyte proliferation induced by allogeneic stimulation. We obtained PBMCs from two distinct common marmosets, with different genetic backgrounds, and co-cultured them as responders and stimulators. We also added M(IL-4) or M(DZNep) to the mixed culture and examined the responder proliferation (Fig. 4a). Without stimulators, responders showed no proliferation, but allogeneic stimulation induced a significant proliferation (Fig. 4b). When M(IL-4) was added, there was no difference from MLR group. However, when M(DZNep) was added, a significant suppression of responder proliferation was observed. Therefore, M(DZNep) but not M(IL-4) could suppress the allogeneic immune response of common marmoset PBMCs.

## Discussion

In this study, we introduce a culture protocol to differentiate macrophage-like cells with immunosuppressive functions from CMESCs. Furthermore, we show that the CMESCs-derived M(DZNep) are capable of suppressing proliferation of alloreactive T cells. Previous studies have showed several differentiation protocols from PSCs into haematopoietic cells in mouse and human. On the other hand, only one report showed a differentiation protocol of the common marmoset PSCs into haematopoietic cells (published by Kurita et al.^40^. In this paper, the authors used enforced expression of Tal1/Scl gene to enhance haematopoietic differentiation. In this study, we have successfully induced differentiation of common marmoset PSCs into haematopoietic cells, especially myeloid cells, without any gene transfer. Our protocol has two differences from that of the previous report. The first difference is related to the use of feeder cells during the differentiation process. While the previous report has utilized feeder cells, our protocol did not. OP9 cells are of mouse origin, and all cytokines produced by this cell line are mouse-reactive. Thus, we considered that the utilization of feeder cells, in particular the mouse OP9 cell line, is not always necessary for the induction of CMESCs differentiation into haematopoietic cells. Second, the previous report has used the basic fibroblast growth factor (bFGF) for the maintenance and culture of ESCs, while we used leukemia inhibitory factor (LIF) in our protocol. LIF can help to maintain pluripotency and to promote self-replication of PSCs,a major characteristic that represents naïve stem cells in rodents such as mouse. On the other hand, bFGF can similarly help to maintain pluripotency and to promote self-replication of PSCs in mammals including human^41^.

Among the naïve and prime stem cells, several differences in the epigenetic methylation and the consequent differential bias have been reported^42^. Common marmoset PSCs are classified as mammal prime stem cells. However, comparing to bFGF-utilizing protocol described by Kurita et al., the status of LIF-maintained ESCs in our protocol is close to the naïve status, and probably more suitable for the differentiation of haematopoietic cells. Together, these two factors might be contributed to the successful differentiation of PSCs into haematopoietic cells without gene transfer in this study. Recently, the same group reported haematopoietic differentiation was promoted by transient PI3K inhibition in CMESCs without any gene transfer^43^. Combining these findings may lead to more efficient induction of CMESCs-derived haematopoietic cells.

For cell culture, bovine fetal serum has been widely used. However, when generating cells for therapeutic purposes in patients, using the bovine fetal serum may be accompanied with abnormal immune responses, and thus should be avoided in clinical applications. The serum replacement has been previously described for the differentiation of dendritic cells from human ESCs^31^. Similarly, serum replacement should be evaluated in the differentiation protocol established in this study. Additionally, evaluation of the efficacy of activating Wnt/β catenin signaling pathway which was previously reported to increase the efficiency of PSCs differentiation into haematopoietic cells^44^ and fine-tuning of cytokine concentrations should be considered in future works for establishing more effective, safe and less-cost differentiation method.

Previously, we have successfully induced differentiation of ES/iPS cells into immunosuppressive macrophages, which were capable of extending survival time of grafts derived from the same ES/iPS cells when implanted into allogenic mice^27,28^. Additionally, we showed that the immunosuppressive functions of these differentiated macrophages were NO-dependent upon the stimulation with IL-4 and LPS. Similarly, we tried the same stimulation in myeloid cell fraction obtained from the differentiation process. However, with IL-4- and LPS-stimulated CMESCs-derived macrophages showed no NO production upon stimulation (Supplementary Fig. 1c).

In this study, the low molecular weight compounds DZNep was used to induce the immunosuppressive phenotype in CMESCs-derived macrophage-like cells (Fig. 2a). In addition to its effects on the acquirement of immunosuppressive phenotype in macrophages^38^^,^ DZNep was reported to be efficient for the generation of iPS cells^45^. Sodium valproate (VPA) is another low molecular weight compounds used to induce a shift in macrophage phenotype into an immunosuppressive status^37^. VPA is known as a histone deacetylase inhibitor, and has been reported as an efficient factor for iPS generation^46^. When combined together, DZNep and VPA involve in reprogramming events, represented by inducing euchromatic changes and promoting the activation of various genes. By reflecting these backgrounds on our established protocol that combine IL-4 with DZNep, the immunosuppressive properties of differentiated cells are expected to be mediated by the effects of IL-4 on chromatin structure resulting in an immunosuppressive phenotype, which is further enhanced by DZNep-induced euchromatic changes. To confirm this, the histone methylation status in the promoter region of genes that show differences between M(IL-4) and M(DZNep) such as PD-L1, IL-10, and TGF-β should be evaluated. However, as far as we investigated, PD-L1 and IL-10 may not contribute to immune suppression by M(DZNep) (Supplementary Fig. 2e). Gene expression of M1- or M2-polarized markers in M(DZNep) was confirmed by RT-PCR and M(DZNep) showed the expression of M2 marker genes, such as *irf4* and *gata3*, suggesting that the combination of IL-4 and DZNep along with the GM- and M-CSF stimulation led the precursor cells to M2-like immunosuppressive phenotype. Thus, further analysis on M(DZNep) including global transcriptomic and epigenetic changes, or knockout of candidate genes are required to understand the effect of DZNep in our differentiation method and M(DZNep) suppressive function. It is also of great interest to examine whether this differentiation protocol can be achieved by the replacement of DZNep with other low molecular weight compounds that can induce similar euchromatic changes or function like VPA.

In recent years, great advance has been achieved in the field of basic research that focuses on ES/iPS cell applications in regenerative medicine. More importantly, some of these applications have found a way into patients in clinic, indicating a rapid translation from basic into clinical research. Under such condition, it is of great importance to focus in pre-clinical experimental models on mammals that are close to human such as common marmoset, rather than rodents or others.

In conclusion, we report a successful differentiation of CMESCs into immunosuppressive macrophages. Future works will focus on the ability of these immunosuppressive macrophages to suppress allogenic immune reaction against grafts derived from the same ES donor in a transplantation model in common marmoset.

## Methods

### Cells, and culture media

Common marmoset ES cells (NO.40) were established previously^23^. ES cells were maintained in ESCs media [Dulbecco's Modified Eagle's Medium/Ham's F-12 with l-glutamine supplemented with 20% of knockout serum replacement (WAKO), 0.1 mM of nonessential amino acids, 0.1 mM of 2-mercaptoethanol, 10 U/mL of penicillin and 100 µg/mL of streptomycin (Nacalai tesque) containing recombinant human leukemia inhibitory factor (in-house)] on feeder layers of irradiated mouse embryonic fibroblasts.

### Differentiation culture

The undifferentiated CMESCs were plated in petri dish at 1 × 10^6^ cells/10 cm dish in 10 ml of α-minimum essential media supplemented with 20% fetal calf serum, 0.1 mM of 2-mercaptoethanol, 0.1 mM of nonessential amino acids, 1 mM of sodium pyruvate, and 10 U/mL of penicillin and 100 μg/mL of streptomycin, 20 ng/mL of SCF, 20 ng/mL of VEGF, 20 ng/mL of BMP4 and 20 ng/mL of GM-CSF to generate embryoid body (EB). During the differentiation process, BMP4 was removed on day 5, VEGF was removed on day 10, and SCF was removed on day 15. On day 20 of culture, adherent cells were obtained as monocytes. For generation of M(IL-4) and M(DZNep), further stimulation for 5 days with 20 ng/mL of GM-CSF, 20 ng/mL of M-CSF, 20 ng/mL of human interleukin 4 and 0.1 µM of DZNep (Cayman chemical). All cytokines were purchased from Biolegend.

### Phagocytosis assay

CMESCs-derived macrophage-like cells were plated in µ-Slide 8 well (ibidi) at 5 × 10^4^ cells/well. pH-sensitive pHrodo green *E. coli* bioParticles (Thermo Fisher Scientific) were opsonized for 24 h in cell culture medium containing 10% FBS. Opsonized particles were added to cell culture and incubated for 120 min. After incubation, the cultured cells were fixed with 4% paraformaldehyde (WAKO), washed with PBS and then stained nuclei with DAPI (Cayman chemical) for 30 min at room temperature. Fluorescence images were acquired using an observer z1 microscope (Zeiss).

### Reverse-transcription and quantitative polymerase chain reaction

RNA was extracted using RNeasy Mini Kit (Qiagen) according to the manufacturer’s protocol. Reverse transcription polymerase chain reaction (RT-PCR) and quantitative PCR (qPCR) reaction were performed using KAPA SYBR Fast qPCR Kit (Nippon Genetics) with the following primers (all for common marmoset genes). *arginase 1*: forward 5′-TTCTCAAAGGGACAGCCACG-3′, reverse 5′-TAGGGATGTCAGCAAAGGGC-3′; *transforming growth factor-β1*: forward 5′-CCCCTACATTTGGAGCCTGG-3′, reverse 5′-CACGTAGTACACGATGGGCA-3′; *interleukin-10*: forward 5′-GGTGCAGGTGAAGAATGCTG-3′, reverse 5′-GAGTCTATGGAGTCGCCGC-3′; *interleukin-6*: forward 5′-GATTCAATGAGGAGACTTGCC-3′, reverse 5′-TGTTCTGGAGGTACTCTAGGTA-3′; *tumor necrosis factor-α*: forward 5′-AGCCTGTAGCCCATGTTGTAG-3′, reverse 5′-CTCTCAGCTCCACGCCATTG-3′; *octamer-binding transcription factor 4*: forward 5′-AAACCCACACTTCAGCAGATCA-3′, reverse 5′-CACACGGACCACATCCTTCTC-3′; *sex determining region Y-box 2*: forward 5′-GAGAACCCCAAGATGCACAAC-3′, reverse 5′-TCTCGGACAGCAGCTTCCA -3′; *irf5*: forward 5′-ACTCTTTGGCCCCATAAGCC-3′, reverse 5′-GGTCTGCCCCTTCATTGAGA-3′; *irf4*: forward 5′-CCAGATCGACAGTGGCAAGT-3′, reverse 5′-TGTCGATGCCTTCTCGGAAC-3′; *il23a*: forward 5′-ACAACAGTCAGTTCTGCTTGC-3′, reverse 5′-CGAGCTGTTGGCTTTAGGGA-3′; *gata3*: forward 5′-CAGAGGTACCCTCCGACTCA-3′, reverse 5′-CCGTGGTGAATGGACGTCTT-3′; *actin-β*: forward 5′-TCCTGACCCTGAAGTACCCC-3′, reverse 5′-GTGGTGGTGAAGCTGTAGCC-3′.

### Flow cytometry and antibodies

Flow cytometry was performed using FC500 instrument (Beckman Coulter) and data were analyzed by FlowJo software (Tree Star). Anti-common marmoset CD45 (6C9), anti-human CD14 (M5E2), anti-mouse/human CD11b (M1/70), anti-human CD86 (2331), anti-human HLA-ABC (G46-2.6), anti-human HLA-DR (L243), anti-human PD-L1 (29E.2A3), Anti-mouse IL-10 (JES5-16E2) and corresponding isotype controls were purchased from Biolegend or Becton, Dickinson and Company. For analysis, live cells were gated based on forward and side scatter as well as lack of propidium iodide uptake. All antibodies were used at 1:150 dilutions.

### Mixed lymphocyte reaction

Common marmoset peripheral blood mononuclear cells were separated by percoll gradient centrifugation and used as responder cells. Responders (1 × 10^5^) were co-cultured with 35 Gy-irradiated stimulators (Allogeneic common marmoset PBMCs, 2 × 10^4^) in 96-well round-bottomed culture plates. For some experiments, 35 Gy-irradiated M(IL-4) or M(DZNep) were added to the culture. After 4 days, the cells were pulsed with [^3^H] thymidine, harvested 8 h later, and measured for uptake of [^3^H] thymidine as previously described. In the experiment for the analysis of dose-dependency, M(DZNep) was added with graded number to MLR culture. Where indicated, following antibodies were added to MLR culture; anti-human IL-10 (Biolegend, clone: JES3-9D7), anti-human PD-L1 (Biolegend, clone: 29E.2A3), Rat IgG (Jackson ImmunoResearch), and Mouse IgG (Biolegend, clone: MOPC-21).

### Statistical analyses

Significant differences were evaluated by using the unpaired Student *t* and Tukey Honest Significant Difference tests. *P* values < 0.05 were considered significant.

## Supplementary information

Supplementary Information.
