# Supplementary material for: Induction of macrophage-like immunosuppressive cells from common marmoset ES cells by stepwise differentiation with DZNep
Source: Sci Rep. 2020 Jul 28;10:12625. doi: 10.1038/s41598-020-69690-9 (PMC7387549; doi:10.1038/s41598-020-69690-9)
Supplement: Supplementary file 1 — Supplementary Information. [file 41598_2020_69690_MOESM1_ESM.pdf]

Induction of macrophage-like immunosuppressive cells from common marmoset ES cells by stepwise differentiation with DZNep

Hyuma Tsuji[1,3], Ryo Otsuka[1,3], Haruka Wada[1], Tomoki Murata[1], Airi Sasaki[1], Mizuho Itoh[1], Muhammad Baghdadi[1], Erika Sasaki[2], Ken-ichiro Seino[1*]

[1]Division of Immunobiology, Institute for Genetic Medicine, Hokkaido University, Kita-15 Nishi-7, Sapporo 060-0815, Japan

[2]Central Institute for Experimental Animals, 3-25-12 Tonomachi, Kawasaki, Kanagawa 21-0821, Japan

[3]These authors contributed equally to this work.

Correspondence

Ken-ichiro Seino, M.D. Ph.D.

Division of Immunobiology, Institute for Genetic Medicine, Hokkaido University, Kita-15 Nishi-7, Sapporo 060-0815, Japan

Tel: +81-11-706-5531, Fax: +81-11-706-7545

E-mail: seino@igm.hokudai.ac.jp

Supplementary Figure 1

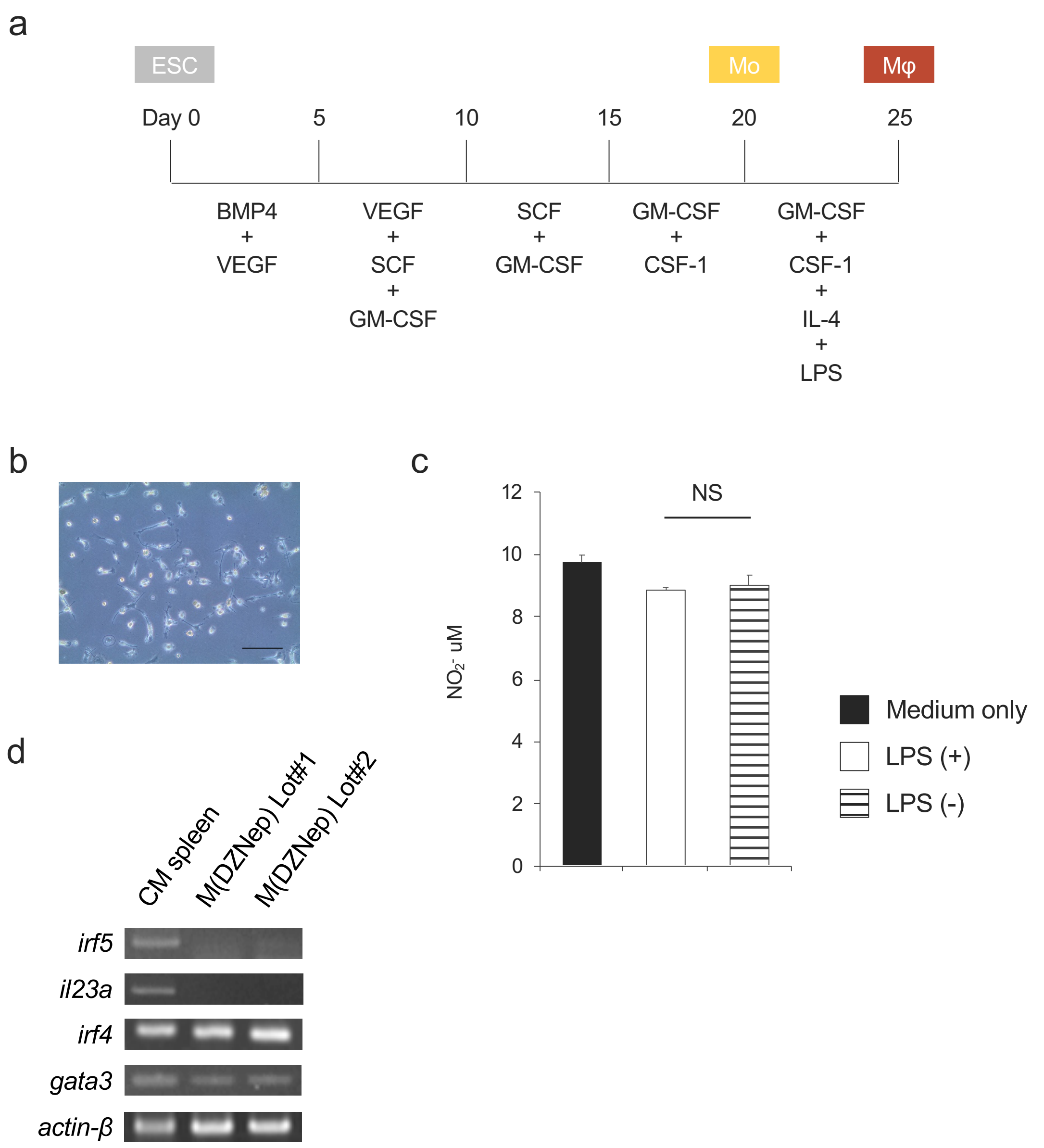

Supplementary Figure 1: LPS-stimulated macrophages showed no immunosuppressive properties
(a) A scheme describes the culture protocol used to obtain CMESCs-derived macrophage-like cells with LPS-stimulation. (b) Transmitted light image of CMESCs-derived macrophage-like cells. Scale bars: 100 µm. (c) Measurement of NO concentration in the supernatants of macrophage-like cells or LPS-stimulated macrophage-like cells. (d) Non-quantitative reverse transcription-PCR analysis for M1 (irf5 and il23a) and M2 (irf4 and gata3) marker genes. Full length gel picture is presented in Supplementary fig. 3. Results are expressed as mean ± SD. NS: no significance. CM: common marmoset.

Supplementary Figure 2

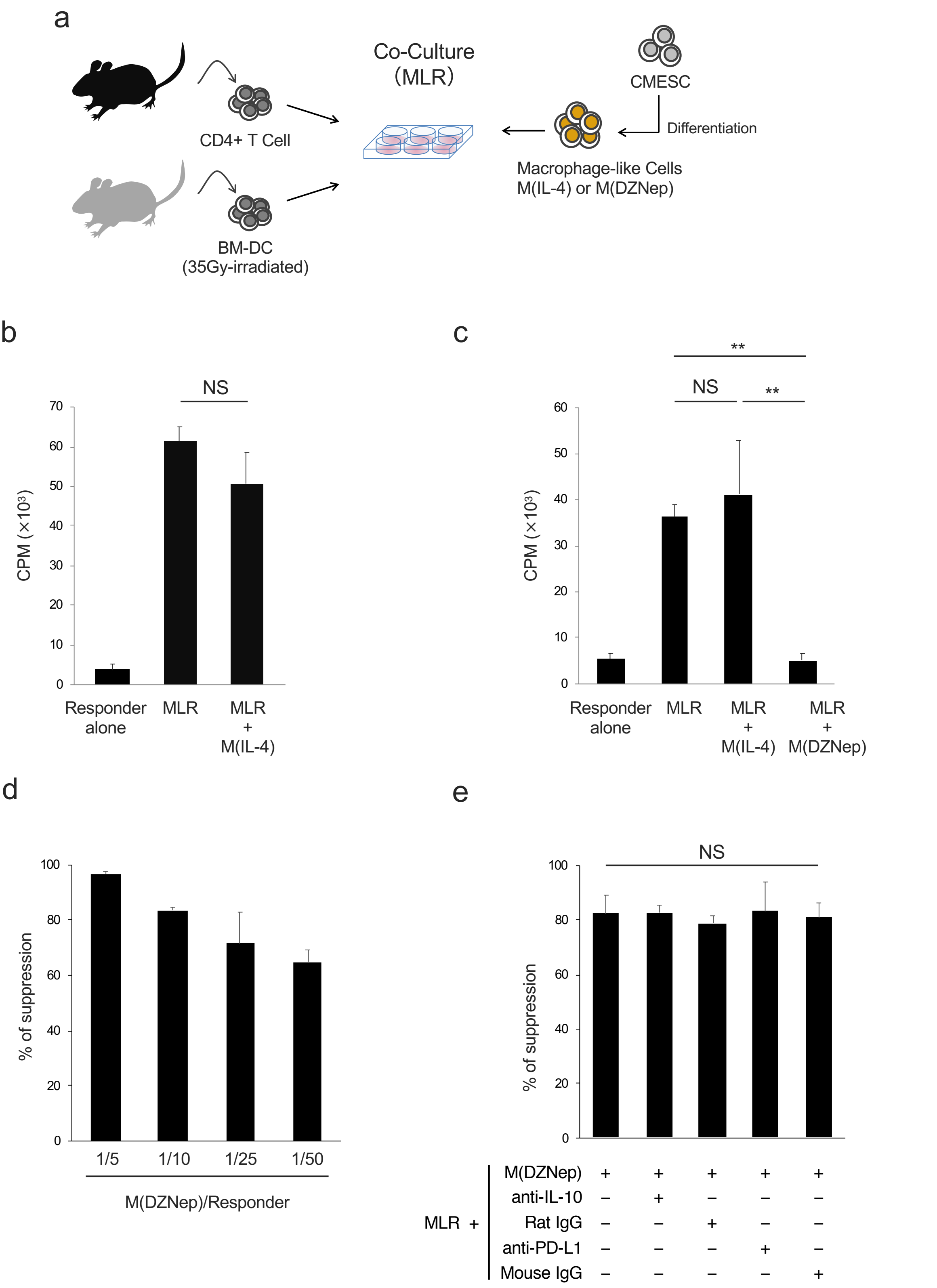

Supplementary Figure 2: M(DZNep) but not M(IL-4) suppress T cell proliferation
(a) M(IL-4) and M(DZNep) were cultured together with allo-proliferative mouse T cells for 4 days. Cell proliferation measured for uptake of [3H] thymidine. (b) MLR assay: 1×105 of CD4+ T cells from C3H mice were stimulated with irradiated BALB/c BM-DCs in direct coculture (5:1). To test the capability to inhibit T cell proliferation, M(IL-4) were added to MLR culture at same numbers of stimulator cells. (c) MLR assay: To test the capability to inhibit T cell proliferation, irradiated M(IL-4) or M(DZNep) were added to MLR culture at same numbers of stimulator cells. (d) MLR assay: To test a dose-effect, graded number of M(DZNep) was added to MLR culture described in Supplementary Fig. 2a. (e) MLR assay: To identify responsible molecules for suppressive effect, several immunosuppressive molecules were blocked by specific inhibitors: 10 µg/ml of anti-IL-10 and anti-PD-L1 monoclonal antibodies, or corresponding isotype-matched controls. Results are expressed as mean cpm or % of suppression ± SD. **P <0.01, NS: no significance.

Supplementary Figure 3

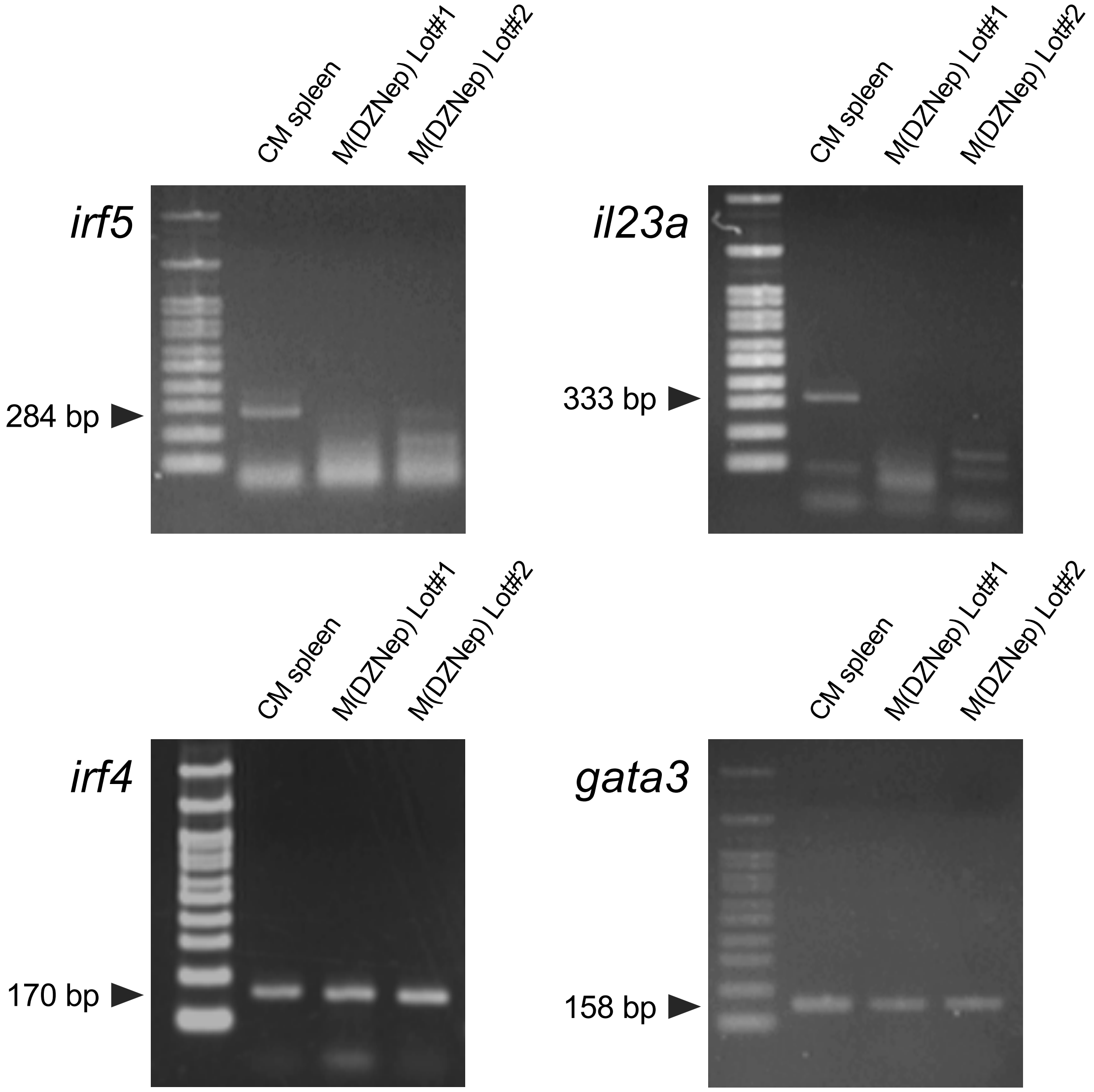

Supplementary Figure 3: Full size gel images of Supplementary Fig. 1d
Agarose gel electrophoresis of RT-PCR products obtained with RNA from common marmoset spleen or M(DZNep). Expected product sizes are indicated with arrowheads. 100 bp ladder size standards were loaded in the leftmost lane.
